# The Role of IL-17-Mediated Inflammatory Processes in the Pathogenesis of Intervertebral Disc Degeneration and Herniation: A Comprehensive Review

**DOI:** 10.3389/fcell.2022.857164

**Published:** 2022-03-03

**Authors:** Kaori Suyama, Daisuke Sakai, Masahiko Watanabe

**Affiliations:** ^1^ Department of Anatomy and Cellular Biology, Basic Medical Science, Tokai University School of Medicine, Isehara, Japan; ^2^ Department of Orthopaedic Surgery, Surgical Science, Tokai University School of Medicine, Isehara, Japan

**Keywords:** interleukin-17, intervertebral disc, nucleus pulposus, disc herniation, cytokine

## Abstract

It has been reported that degenerated and herniated lumbar intervertebral discs show high expression of IL-17, suggesting that local immune reactions occur in patients with low back pain. While clinical sample analyses from different laboratories confirm this, it is not deeply not known on how IL-17 is induced in the pathology and their interactions with other inflammatory responses. This conscience review organizes current laboratory findings on this topic and present trajectory for full understanding on the role of IL-17 in pathology of intervertebral disc disease.

## Introduction

The intervertebral disc (IVD) consists of an outer fibrocartilaginous annulus fibrosus (AF) that surrounds a gel-like nucleus pulposus (NP). Its main functions are to act as a shock absorber and maintain the backbone mobility, including the cartilaginous endplates that cover this assembly on both the top and bottom sides. The AF is characterised by 15–25 concentric lamellae consisting of fibres of collagen types I and II, with smaller amounts of collagenⅢ, proteoglycans and elastin (Urban and Roberts, 2003; Önnerfjord et al., 2012). A small number of capillaries that penetrate only a few millimetres into the outermost AF (Sakai and Grad, 2015). NP is an avascular tissue. NP is comprised mainly of water along with collagen fibrils (including types VI, IX, and XI), various proteoglycans for shock absorption, and cells of the NP, which are adapted to survive in this hypoxic environment (Urban and Roberts, 2003; Hiyama et al., 2015; Sakai and Grad, 2015). Due to its avascular nature, the nutrients and metabolites are exchanged by diffusion to and from microvessels in the cartilaginous endplates and outer AF (Vo et al., 2016).

With aging, trauma, genetic susceptibility, and other factors, the IVDs gradually degenerate due to many factors, such as microenvironment changes and cell death (Zhang F et al., 2016). During IVD degeneration, the structure of the disc changes and homeostasis in disc become disturbed (Sakai and Grad, 2015). IVD degeneration is linked to low back pain and sciatica, which lead the physical disability of the patients (Urban and Roberts, 2003). Current studies demonstrate that IVD degeneration progresses in consequence of many factors, such as biomolecular damage, inflammatory response, IVD cell loss, extracellular matrix (ECM) degradation increase, and synthesis reduction (Vo et al., 2016; Zhang F et al., 2016).

The frequently reported inflammatory cytokines that are secreted and promote IVD degeneration include tumor necrosis factor-α (TNFα), IL-1 α/β, IL-6, IL-17, IL-8, IL-2, IL-4, IL-10, COX-2, IFN-γ, chemokines, and prostaglandin (PGE)2. Moreover, the factors promoting the extracellular matrix (ECM) degradation often reported are matrix metalloprotease (MMP)-1, -3, -7, -9, and -13, and A disintegrin-like and metalloprotease with thrombospondin type-1 motif (ADAMTS)-1, -4, -5, -9, and -15 (Risbud and Shapiro, 2014; Vo et al., 2016; Willems et al., 2016; Zhang F et al., 2016; Sutovsky et al., 2017). Conversely, TGF-β is essential in maintaining IVD homeostasis (Risbud and Shapiro, 2014; Chen et al., 2019).

Recently, high levels of IL-17A were associated with IVD degeneration (IDD) and IVD herniation (LDH) and IL-17A is considered as the crucial factor in IVD pathology (Shamji et al., 2010; Gabr et al., 2011; Cheng et al., 2013; Suyama et al., 2018). Furthermore, the rat tail suspended or punctured model showed the expression of IL-17A in NP cells and AF cells with tissue degeneration or injury (Han et al., 2015; Wang et al., 2018; Ding and Li, 2020). In this review, based on the current literature, we will focus on the IL-17 signaling pathway, its interaction with other factors, and multiple functions in IVD pathology.

## Production of IL-17

IL-17 (IL-17A, CTLA8) cDNA was isolated and cloned from murine hybridomas in 1993 (Rouvier et al., 1993). IL-17 (also called IL-17A) is produced by the T helper 17 (Th17), a subset of CD4^+^ T cells that are distinct from classic Th1 and Th2 lineages (Gaffen, 2011; Iwakura et al., 2011). These Th17 cells are characterized by the expression of the “master” transcription factor RAR-related orphan receptor gamma (RORγt) and are activated by the IL-12 family cytokine IL-23 (McGeachy et al., 2019). The IL-17A producing CD4^+^ Th17 cells arise from multiple differentiation triggers, including TGFβ, IL-6, IL-1β, and IL-21 (Gaffen, 2011). Besides IL-17A, IL-17 consists of six other family molecules (IL-17B, IL-17C, IL-17D, IL-17E (IL-25), and IL-17F) with structural identity. IL-17A and IL-17F are closely related linked genes that are usually coproduced by Type 17 cells (Aggarwal and Gurney, 2002).

It was reported that Th17 cells are the major source of IL-17A, and other innate immune cells produce IL-17A in response to pathogens or tissue injury (Cua and Tato, 2010). Overall, IL-17A is produced from Th17 cells and other innate immune cells, inducing various products, including cytokines (IL-6, granulocyte-colony-stimulating factor [G-CSF], TNFα), chemokines (CXCL1, CXCL2, CCL20, among many others), inflammatory effectors (acute-phase proteins, complement), and antimicrobial proteins (defensins, mucins) (Onishi and Gaffen, 2010).

Recently, numerous studies in humans and mice have suggested that IL-17A plays a leading role in the pathogenesis of different immune-mediated diseases, including rheumatoid arthritis, psoriasis, asthma, and inflammatory bowel disease (Cua and Tato, 2010; Onishi and Gaffen, 2010; Gaffen, 2011; Amatya et al., 2017).

### Th17 and IL-17A in IVD

In patients with lumbar IDD, the percentage of Th17 that are the main source of IL-17A and IL-17A expression in peripheral blood, demonstrated significant increase (Zhang et al., 2014). Furthermore, in LDH, which NP herniated with AF rupture, compared with the healthy controls, the elevated levels of Th17 lymphocytes and IL-17A correlated with the patients’ pain intensity of sciatica, suggesting that the rupture of the AF and herniation of the NP are initiators of an autoimmune response to a ruptured lumbar disc (Cheng et al., 2013). Interestingly, the Th17 cell frequency and IL-17A concentration positively correlated with the visual analog scale score of low back pain and PGE2 expression levels (Zhang et al., 2014).

Many reports have shown that the cytokines trigger Th17 to produce IL-17A in IVD. The Th17 cells producing IL-17A arise from CD4^+^ Th17 cells by stimulating cytokines, such as IL-6, and they secrete IL-17A in IDD and LDH pathologies. When Shamji et al. analyzed IDD and LDH patients’ samples, the percentage of CD4^+^ lymphocytes and CD68^+^ macrophages were significantly higher in NP of both IDD and LDH compared with healthy IVD, and the expression of IL-4, IL-6, IL-12, IL-17, and IFNγ were significantly higher in NP of LDH compared with IDD; notably, IL-17A was particularly elevated. These findings suggest that Th17 differentiation and activation inducing IL-17A production mediate the inflammatory processes underlying IVD pathology (Shamji et al., 2010). Similarly, high IL-6, IL-17A, and TNFα levels were observed in the serum of lumbar radiculopathy patient group compared with the neuropathic pain group, and Th17 was higher in the venous blood of lumbar radiculopathy patients group compared with the neuropathic pain group (Shamji et al., 2017).

Further, IL-23 (Jiang et al., 2016; Schinocca et al., 2021) and IL-21 (Cua and Tato, 2010) were reported as the cytokines stimulating Th17 to produce IL-17A in IDD or LDH. IL-23 expression was significantly increased in human LDH tissues, and it showed significant positive correlations between IL-23 and IL-17A expression. Therefore, the canonical inflammatory-related signaling IL-23/IL-17A axis may play a critical role in degenerated IVD (Jiang et al., 2016). Moreover, as an illustration of the IL-17A and IL-21 correlation, LDH patients exhibited significantly higher serum IL-21 and IL-17 than healthy controls (Xue et al., 2015).

### Th17 and CC Chemokine

There are many studies on the relation between Th17 and chemokines. Th17-related cytokines such as IL-17A, IL-22, and TNFα increased the expression of CC chemokine ligand (CCL) 20 and its only receptor, the CC chemokine ligand-receptor (CCR)6, in human keratinocytes (Harper et al., 2009). Th17 cells predominantly express CCR6 and produce CCL20 as its ligand in rheumatoid arthritis models (Hirota et al., 2007). CCR6 is specifically expressed on the surfaces of Th17 cells, and it is associated with Th17 infiltration (Liu and Rohowsky-Kochan, 2008; Pene et al., 2008). A study of human T cells revealed that CD4^+^CD45RO^+^CCR6^+^ cells contain and secrete much more IL-17A mRNA and more IL-17 protein than CD4^+^CD45RO^+^CCR6^−^ cells (Singh et al., 2008).

The studies analyzing CCL20-CC6 in the disc tissues of IVD disease patients reported that IL-17A-producing cells (CD4^+^IL-17A^+^ and CD4^+^CCR6^+^) appeared in the NP tissues if the AF was ruptured. Furthermore, these studies revealed that NP cells could produce abundant CCL20 and that Th17 associated cytokines (IL-17A and TNFα) can upregulate CCL20 production (Zhang et al., 2013). It was also reported that TNFα stimulation contributes to the gene expression level of *CCL20* in IDD cells (Liu et al., 2015; Wang et al., 2020).

In an *in vivo* study, the expression levels of CCL20, IL-17A, and CCR6 of IVD tissues were dramatically elevated compared with the control groups and needle punctured disc groups in the IVD degenerated model that the needle punctured NP tissue grafted on the nerve root. Furthermore, using ELISA, it was shown that the circulating IL-17A in the serum was elevated in the same models (Zhang Y et al., 2016).

The analysis of the correlation between chemokines and inflammatory cytokine gene expression in humans reported a significant correlation between CCR6 and IL-17A expression both in IVD tissues and blood samples (Singh et al., 2008; Hiyama et al., 2021). These studies indicate that IL-17A-producing cells might participate in the degeneration of disc tissues *via* an interaction with the CCL20/CCR6 system *in vivo*.

## IL-17A Receptor

The IL-17 receptor family includes five members (IL-17RA to IL-17RE) that have such conserved structural characteristics as extracellular fibronectin III-like domains and cytoplasmic expression similar to that of fibroblast growth factor genes and IL-17Rs, and Toll-IL-1R family (SEFIR) domains (Iwakura et al., 2011; Gu et al., 2013).

**FIGURE 1:**
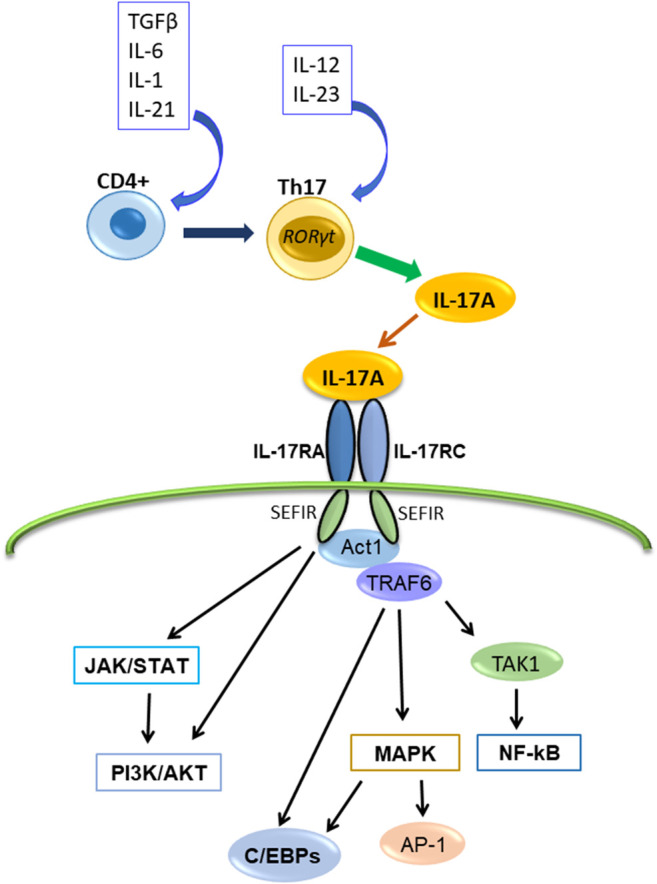
The production of IL-17A, its receptor and transduction signaling pathways. IL-17A is produced by Th17cell and binds the receptor composed of IL-17RA and IL-17RC, and interacts with Act1. Subsequently, Act1 activates multiple independent signaling pathways.

IL-17A binds the heterodimeric receptor complex composed of IL-17RA and IL-17RC (Toy et al., 2006; Gu et al., 2013) (Figure 1) The initial event in IL-17A receptor signaling is recruitment of NF-κB activator 1 (Act1), a multifunctional signaling protein that also contains a SEFIR domain necessary for IL-17A receptor-Act1 association (Gu et al., 2013; McGeachy, et al., 2019). Then, IL-17A signaling activates several intracellular pathways or factors. For example, the IL-17A signaling activates Activator protein-1 (AP-1), TNF receptor-associated factor (TRAF)6, NF-κB, Jun N-terminal kinase (JNK), Erk1/2 and p38, CCAAT/enhancer-binding proteins (C/EBPs), Janus kinase (JAK) and phosphoinositol-3 kinase (PI3K). It also induces several proinflammatory cytokines (including IL-1β, IL-6, TNFα, and CCL2), antimicrobial peptides (β-defensin), and matrix metalloproteinases (Huang et al., 2007; Gaffen, 2009; Gaffen, 2011; Amatya et al., 2017; Chen et al., 2019; Schinocca et al., 2021).

### IL-17A Receptor of IVD Cell

There are many reports based on the addition of IL-17A to culture NP cells showing that IL-17A affects NP cells directly *in vitro* (Gabr et al., 2011; Lin et al., 2015; Wang et al., 2015; Hu et al., 2016; Li et al., 2016; Yao et al., 2016; Suyama et al., 2018; Liu et al., 2019; He et al., 2020). In an *in vitro* study, IL-17A treatment of NP cells isolated from IVD revealed that IL-17A inhibits the proliferation of cells and extracellular matrix synthesis (Lin et al., 2015). Furthermore, treatment with IL-17A and anti-IL-17A neutralizing antibodies caused a significant decrease in the response of IL-6, COX-2, MMP-3, and MMP-13. The small-molecule compounds identified as inhibitors by binding to the IL-17A-binding region of IL-17R by *in silico* analysis revealed effects similar to the evaluation of the IL-17A-neutralizing antibody (Suyama et al., 2018). According to these studies, NP cells have an IL-17A receptor (IL-17R) on their cell surface, IL-17A could affect intracellular reactions by forming an IL-17A/IL-17R complex, and IL-17A signaling participates in IVD pathology.

## IL-17A Signaling

The major downstream pathway of IL-17A signaling is NF-κB pathway (Xie et al., 2010; Gu et al., 2013). NF-κB activator 1 (Act1) contains a SEFIR domain and TRAF6 binding motif (Chang et al., 2006; Li et al., 2007). After IL-17A binding to IL-17R (IL-17RA/IL-17RC), Act1 interacts with IL-17A receptor through the SEFIR domain, and TRAF6 interacts with the TRAF6 binding motif of Act1; subsequently, TRAF6 activates the transforming growth factor β-activated kinase (TAK)1 and the inhibitor of NF-κB kinase (IKK) complex composed of IKKα, IKKβ, and IKKγ, and then, NF-κB activation occurs (Chang et al., 2006; Li et al., 2007; Amatya et al., 2017) (Figure 1). Because it was reported that IL-17A alone is insufficient for strong activation of NF-κB, IL-17A synergizes with other cytokines like TNFα to stimulate NF-κB and enhances the stabilization of proinflammatory cytokine and chemokine mRNA expression (Hartupee et al., 2007; Onishi and Gaffen, 2010; Gu et al., 2013).

Moreover, IL-17A–IL-17R–TRAF6 can promote the activation of the mitogen-activated protein kinase (MAPK) pathway and AP-1 (Walsh et al., 2015). And IL-17A stimulation recruits TRAF4, which binds the same binding site of TRAF6 on Act-1 competitively (Zepp et al., 2012; Gu et al., 2013). Act1-TRAF4 interaction specifically directs the activation of the MEKK3–MEK5–ERK5 cascade and results in MAPK pathway activation (Wu et al., 2015). Additionally, TRAF3 binds directly to the IL-17RA and interferes with IL-17RA-Act1-TRAF6 interaction (Zhu et al., 2010). These reports show that IL-17A activates the canonical NF-κB pathway but not the noncanonical pathway.

### NF-κB Pathways

NF-κB signaling pathways play a crucial role in the pathophysiology of IVD degeneration (IDD) (Sakai and Grad, 2015; Zhang G. Z et al., 2021). The activation of the NF-κB pathway releases of inflammatory cytokines, such as TNFα, IL-2, IL-6, INF-γ, and catabolic enzymes, including matrix metalloproteinase (MMP)-3, MMP-9, MMP-13, ADAMTS-4, and ADAMS-5 (Zhang G. Z et al., 2021). These IL-17A-induced factors contribute to the progression of IDD and LDH pathology.

**FIGURE 2:**
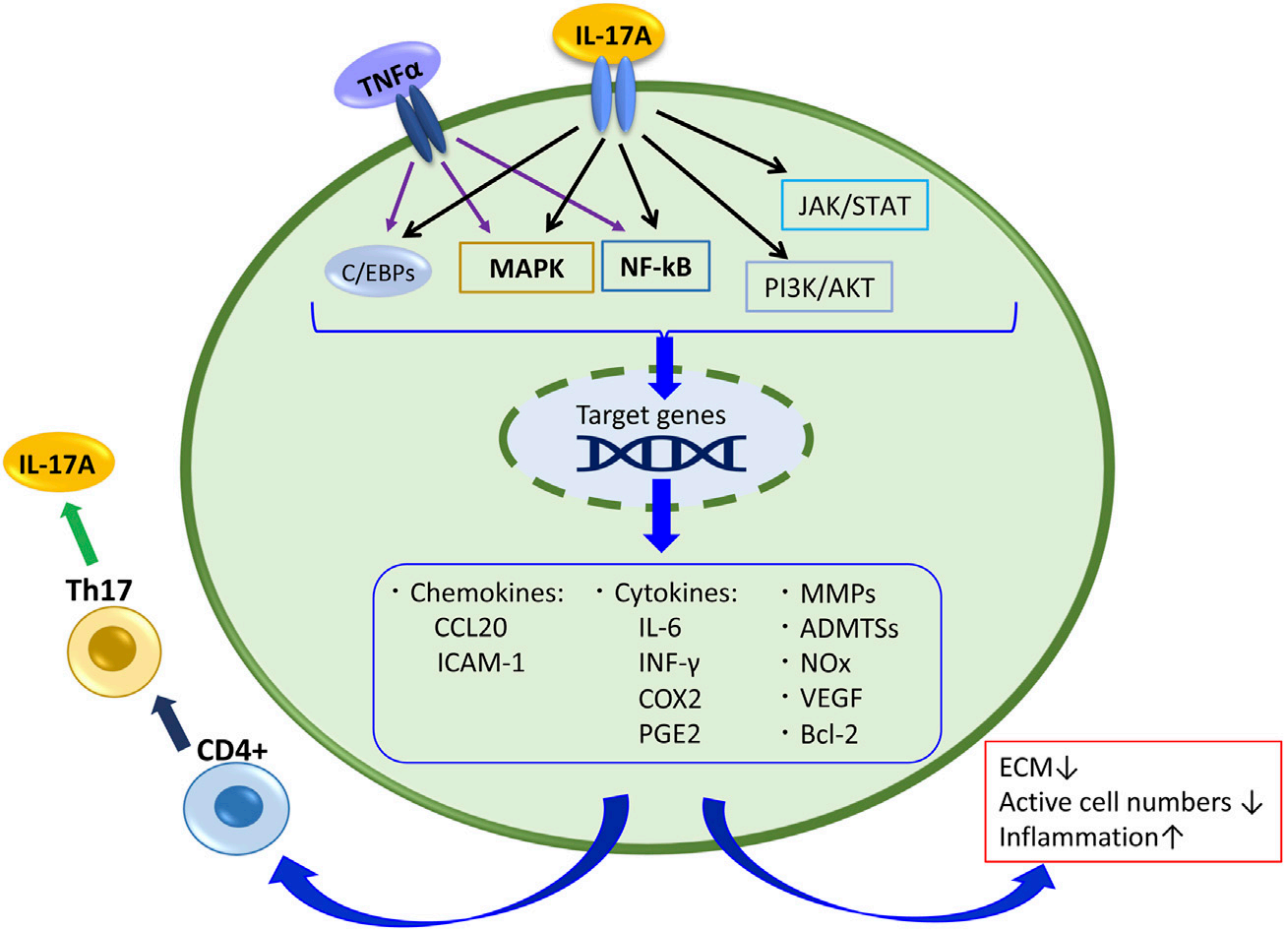
IL-17A signaling and its related products in disc cell and tissue. Also,TNFα stimulates the NF-kB and MAPK pathways and C/EBPs, and contributes to the production of cytokines, chemokines, and extracellular matrix-degrading factors in IVD.

A study analyzing the samples of NP tissue obtained from the IDD patients by the surgical intervention reported that the expression levels of IL-17A and TNFα in the discs of the AF-disrupted group were significantly higher than those in the AF intact group, and the IL-17A and TNFα expression levels were correlated (Wang et al., 2015; Liu et al., 2016). Furthermore, ADMTS-7 expression levels were significantly elevated with a degenerative grade of discs collected from degenerative patients with IVD. Etanercept, an antagonist of TNFα, decreased the expression of ADMTS-7 induced by IL-17A in NP cell culture evaluations (Wang et al., 2015). Yao et al. reported that IL-17A increased the production of MMP-13 and decreased expression of collagen type II alpha 1 (COL2A1) and Aggrecan (ACAN) *via* the NF-κB pathway in NP cells (Yao et al., 2016). Similarly, peroxisome proliferator-activated receptor γ (PPAR-γ), which inhibits the NF-κB signal pathway, decreased in degenerative IVD tissues compared with nondegenerated tissues, and a PPAR-γ agonist downregulated the production of IL-1β, CCL20, COX-2, PGE-2, MMP-13, and ADAMTS-7 induced by IL-17A along with TNFα in NP cells (Liu et al., 2019). Overall, these studies indicate that IL-17A synergize with TNFα to stimulate the NF-κB signaling pathway and contributes to the production of cytokines, chemokines, and extracellular matrix-degrading factors in IVD (Figure 2).

### MAPK Pathways

The effects of the MAPK pathways, including three major MAPK, p38 kinase, JNK, and extracellular signal-regulated kinase (ERK), play a vital role in IDD (Zhang F et al., 2016; Zhang H. J et al., 2021). IL-17A signaling mediates the MAPK pathway in NP cells, and IL-17A increased COX-2 and PGE2 production by activating the p38/c-Fos and JNK/c-Jun signaling pathways in an AP-1-dependent manner (Li et al., 2016). Similarly, IL-17A increased IL-6 expression *via* p38 activation in NP cells (Suyama et al., 2018). AP-1 is a downstream transcription factor of the MAPK pathway, and it regulates the expression of several genes, including COX-2 and IL-6, cell proliferation, apoptosis, and inflammation (Shaulian and Karin, 2002; Ye et al., 2014; Yoon and Park, 2021). AP-1 protein is primarily regulated at the level of both Jun and Fos gene transcription involving MAPK pathways and by post-translational modifications *via* phosphorylation and dephosphorylation, and the complex of c-Jun with c-Fos are the members of the AP-1 family of transcription factors (Shaulian and Karin, 2002; Ye et al., 2014). These reports indicate that MAPKs are the IL-17A-induced signals that cause disc cell degeneration. Additionally, a chemokine N-acetylated proline-glycine-proline (N-Ac-PGP) was reported to induce proinflammatory cytokines, including IL-17A and matrix catabolic enzymes in NP cells *via* both NF-κB and MAPK signaling pathways (Feng et al., 2017). These observations suggest that both NF-κB and MAPK pathways involve IL-17A transcription and production in NP cells.

Meanwhile, there are different reports about the role of c-Jun and AP-1 for IDD. Lei et al. reported that c-Jun overexpression upregulated the expression levels of TGF-β, TIMP-3, and COL2A1 in the mRNA and proteins, but simultaneously downregulated the expression levels of inflammatory factors IL-1β, IL-17, IL-6, and TNFα both *in vivo* and *in vitro* studies (Lei et al., 2020; Lei et al., 2021). The downregulation of IL-17A expression and NP cells decreases IL-17A secretion from NP cells and its effects on IL-17A signaling. Furthermore, a more detailed investigation about the relation of IL-17A-MAPK-c-Jun is required.

### CCAAT/Enhancer-Binding Proteins

In addition to the NF-κB and MAPK pathways, IL-17A signaling stimulates the transcription factor CCAAT/enhancer-binding proteins (C/EBPs). IL-17A-IL-17R-Act1-TRAF6 are required for the activation of C/EBPβ and C/EBPδ (Gaffen, 2009; Chang and Ding, 2011; Iwakura et al., 2011). Both NF-κB and C/EBPs transcriptional factor binding sites are over-represented in promoters of IL-17A target genes (Shen et al., 2006). C/EBPs binds to the IL-1β response element in the IL-6 promoter region (Akira et al., 1990), and IL-17A collaborates with TNFα to regulate C/EBPδ (or the related transcription factor C/EBPβ) involved in IL-6 gene expression (Ruddy et al., 2004). On the other hand, a negative regulation of C/EBPs mediated through IL-17RA has been reported. IL-17RA can be mediated through ERK and glycogen synthase kinase 3β (GSK-3β)-dependent mechanisms to phosphorylate C/EBPβ, leading to suppression of the cytokines induced by IL-17RA (Shen, et al., 2009; Chang and Ding, 2011).

With regards to the IVD, both human and rat NP cells expressed C/EBPβ, and C/EBPβ acts as a potent proinflammatory mediator by inducing the TNFα expression levels *via* the ERK1/2 and p38 pathways in rat NP cells in IDD (Hiyama et al., 2016). These reports show that IL-17A can participate in the various responses with TNFα, IL-6, and IL-1β in IVD pathology.

### JAK/STAT and PI3K/AKT Pathways

JAK/STAT and PI3K/AKT pathways are reported as IL-17A mediates and affects several factors in disc cells (Gu et al., 2011; Lee et al., 2013; Hu et al., 2016). IL-17A activates the JAK/STAT pathway by mediating a rapid tyrosine phosphorylation of the JAK family (Tyk 2, JAK 1,2, and 3) and STAT 1, 2, 3, and 4 (Subramaniam et al., 1999). In addition, the activation of JAK by IL-17A induces PI3K/AKT activation (Huang et al., 2007).

A study on IVD revealed that vascular endothelial growth factor (VEGF) and IL-17A are highly expressed in human degenerated NP tissue, and IL-17A upregulated VEGF expression through the JAK/STAT pathway in NP cells (Hu et al., 2016). VEGF contributes to NP cells survival under hypoxic conditions because of the avascular environment of the disc (Fujita et al., 2008). Although under IVD degeneration conditions, some cytokines, including IL-17A, elevated VEGF, which then promoted angiogenesis and vasculogenesis in disc lesions to exacerbate IDD (Lee et al., 2011; Studer et al., 2011; Binch et al., 2014).

With regards to the relation between IL-17A and PI3K/AKT, He et al. reported that IL-17A increases the activation levels of the PI3K/AKT pathway, which then induces the Bcl-2 expression in NP cells, and that led to the suppression of NP cells autophagy (He et al., 2020). In NP tissues, it was reported that autophagy regulates the catabolic effects and apoptosis in inflammatory conditions (Shen et al., 2011; Xu et al., 2015). Therefore, these findings that IL-17A-PI3K/AKT activation inhibits autophagy might lead the progression of IVD degeneration (Shen et al., 2011; Xu et al., 2015).

## The Interaction of IL-17A and Other Cytokines in IVD Cells

In the NP cells from IDD patient’s tissue, the production of NOx, PEG2, IL-6, and ICAM-1 was upregulated by the synergy of IL-17A and TNFα or IFNγ (Gabr et al., 2011). Similarly, it was reported that IL-17A interacts with many cytokines, such as TNFα or chemokines in IVD cells (Shamji et al., 2010; Wang et al., 2015; Zhang Y et al., 2016; Liu et al., 2019). As mentioned above, IL-6 is necessary for Th17 differentiation to produce IL-17A, and it was also expressed by IL-17A signaling in IVD cells *via* MAPK pathways (Suyama et al., 2018). Furthermore, IL-1β was reported to induce IL-17A expression in IDD (Johnson et al., 2015; Wang et al., 2020).

Regarding the cooperation of IL-17A and TNFα in IVD pathology, besides the IL-17A receptor- NF-κB pathway, the TNF receptor TNFR1 and TNFR2 are involved in the IL-17A response. TNFR1 can be activated either by tmTNFα (transmembrane TNFα) or sTNFα (a soluble form), whereas TNFR2 is activated mainly by sTNFα. Under tmTNFα and sTNFα stimulation, the TNFR1/SODD complex releases the inhibitory SODD protein, and TNFR1 becomes activated. Then TNFR1 binds TNF receptor-associated death domain, recruiting other adaptor proteins, including TNF receptor-associated factor 2 (TRAF2), receptor-interacting protein-1 (RIP-1), and cellular inhibitor of apoptosis protein (cIAP) 1, resulting in the formation of complex I that signal through either the NF-κB or MAPK pathways to activate p65 or AP-1 (Johnson et al., 2015; Wang et al., 2020). TNFR2 recruits TRAF3, TRAF2, cIAP1/2, and TRAF1 to establish a complex that also activates NF-κB, AP-1, and ERK, and it activates PI3K/AKT consequently (Wang et al., 2020). This regulation concerns many responses of IL-17A in IVD cells. Further, these pathways regulate the production of pro-inflammatory mediators such as TNFα, IL-1β or IL-6, and these mediators can recruit Th17 cells, which produce IL-17A, again (Figure 2). Indeed, when the factor that inhibits TNFα-TNFR1 signaling was suppressed in murine models of IDD, IL-17A expression was significantly increased by TNFα stimulating the NF-κB and MAPK pathways *via* TNFα receptors (Wang et al., 2018). TNFα can stimulate IVD cells directly, and it affects IL-17A signaling. Therefore, it results in IDD progression.

## Discussion

IL-17A plays an important role in IDD and LDH. IL-17A regulates many factors, including inflammatory cytokines, chemokines, PGE2, and the factors promoting ECM degradation, consequently promoting IVD pathology. However, there are many unknowns in IL-17A about their function, interaction, and gene regulation without immune cells or antagonists. It has yet been unclear how IL-17A is produced from IVD cells, though, it was reported that exposing degenerated disc cells to IL-1β or TNFα stimulates IL-17A production (Yang et al., 2015; Wang et al., 2020). These reports indicate the possibility that several cells can produce IL-17A in disc tissue. Similarly, Risbud and Shapiro suggested that resident disc cells may contribute to the production of lymphokine, including IL-17A (Risbud and Shapiro, 2014) because Shamji et al. showed the prominent IL-17 staining in non-degenerate control tissues (Shamji et al., 2010).

NF-κB and MAPK pathways which IL-17A effected on, regulate pro-inflammatory mediators such as TNF-α, IL-1β or IL-6, as both pathways have been identified as master regulators of inflammation and catabolism in IDD and LDH (Sakai and Grad, 2015). Furthermore, other cytokines or chemokines may be involved in IL-17A functions. Therefore, IL-17A could be used for therapy targets of IVD diseases.

According to current reports, IL-17A receptors are at the surface of NP cells, it indicates that IL-17A inhibitor, such as biologicals or small molecule compounds likely to be efficacious against IDD. These findings show the practical utility of IL-17A inhibitor. The therapy of IL-17A regulation may effect on pro-inflammatory cytokines including TNFα, IL-6, and IL-1β, and may suppress the degradation of ECM. Further, it was suggested the possibility that IL-17A correlates with IVD disease patients’ pain intensity, IL-17A is expected to be a biological marker of curative effects. It suggests the possibility that IL-17A inhibitors can be used as the medication for IDD to suppress the inflammation and the degradation of ECM by improving the IVD specific microenvironment *via* the related pathways in NP cells. It may lead to the improvement of physical functioning and quality of life of IDD patients. Indeed, as the reports of IL-17A medication in other diseases, it was reported that the treatment with IL-17A blockers improved joint symptoms of psoriatic arthritis compared with placebo (Mease et al., 2017; Nash et al., 2017; Blauvelt and Chiricozzi. 2018).

Additionally, although only IL-17A has been associated with IVD, the IL-17 family contains six members (IL-17A–IL-17F) (Iwakura et al., 2011; McGeachy et al., 2019). Therefore, the involvement of additional members of the IL-17 family in IVD could be reported in the future.
